# On the sunny side of (new) life: Effect of sunshine duration on age at first reproduction in Japanese macaques (*Macaca fuscata*)

**DOI:** 10.1002/ajp.23019

**Published:** 2019-06-27

**Authors:** Lena S. Pflüger, Katharina E. Pink, Anja Böck, Michael A. Huffman, Bernard Wallner

**Affiliations:** ^1^ Department of Behavioural Biology University of Vienna Vienna Austria; ^2^ Austrian Research Center for Primatology Ossiach Austria; ^3^ Department of Evolutionary Anthropology University of Vienna Vienna Austria; ^4^ Family and Population Studies Katholieke Universiteit Leuven Leuven Belgium; ^5^ Primate Research Institute Kyoto University Inuyama Aichi Japan

**Keywords:** meteorological conditions, puberty onset, reproductive timing, sexual maturation, sunbathing

## Abstract

To produce offspring early in life is energetically demanding and depends greatly on environmental conditions. In female primates, age at first reproduction (AFR) has been associated with social parameters (e.g., population density and social rank), food availability and meteorological conditions (e.g., photoperiod, rainfall patterns, and temperature). Regarding the latter, less attention has been given to the influence of sunshine. In nonhuman primates, including the northern‐most distributed Japanese macaque (*Macaca fuscata*), sunbathing is an effective thermoregulatory strategy to maintain sufficient energy intake during harsh winter months. Furthermore, the energetic value of sunshine and its role in the synthesis of essential vitamins important for sexual development and overall fertility is well investigated using human and animal models. In the present study, we hypothesized that female's AFR is influenced by the amount of sunshine in a semi‐free‐ranging, provisioned a group of Japanese macaques. To test this, we gathered data on sunshine duration in the year females theoretically experienced the onset of puberty. This phase of the female life cycle is particularly prone to the effects of environmental conditions. In addition to the investigation of sunshine duration and other meteorological conditions (i.e., rainfall and temperature) we controlled for social parameters (i.e., group size and sex ratio) as potential covariates. We found a clear effect of sunshine duration on female AFR: Females who entered puberty in years with more sunshine reproduced for the first time at significantly younger ages than females who experienced less sunshine during this specific period of their development. Possible mechanisms for how the sunshine influences sexual maturation in Japanese macaques are discussed.

## Research highlights

- We investigated the influence of sunshine during the year of puberty onset on a female's age at first reproduction (AFR).

- We used 20 years of birth data from a semi‐free‐ranging group of Japanese macaques and meteorological data provided online by local weather stations

- We controlled for the influence of other meteorological conditions as well as for group size and socionomic sex ratio

- Higher mean sunshine duration in the year of puberty onset significantly decreased females’ AFR.

## 1 INTRODUCTION

Reproduction is a fundamental feature of all living species and is the key to their survival. To produce offspring and to secure their survival, however, is challenging and largely susceptible to social and environmental conditions. An individual harvests energy from the environment over its lifetime and invests it in certain age‐specific life functions such as growth, maintenance, and reproduction (Roff, 2002; Stearns, 1992). In vertebrate populations, a high investment in early‐life reproduction pays off in terms of increased follow‐up fecundity and offspring survival and is thus positively associated with lifetime reproductive success (Hayward, Mar, Lahdenperä, & Lummaa, 2014). Nonetheless, studies on mammals have demonstrated the energetic costs of gestation and lactation in terms of early reproduction and how it affects female fitness (Blomquist, 2012). For example, in female macaques, the investment to produce offspring early in life comes at a cost of reduced lifespan (Blomquist, 2009a). According to life history theory, these trade‐off decisions between somatic and reproductive effort are influenced by ecological conditions (Promislow & Harvey, 1990). Consequently, the affordability of young age at first reproduction (AFR) may depend on environmental circumstances. These involve social factors such as social density and social rank (Koyama, Takahata, Huffman, Norikoshi, & Suzuki, 1992) but also food availability and climatic circumstances that affect a female's physiological condition related to given energy resources (Bercovitch & Strum, 1993; Garcia, Huffman, Shimizu, & Speakman, 2011; Hamada, Hayakawa, Suzuki, Watanabe, & Ohkura, 2003; Muroyama, Kanamori, & Kitahara, 2006). Among many seasonally breeding macaques, most fertile menstrual cycles occur between October and December—a period in which physiological and behavioral changes are energetically demanding and environmental conditions also come into play (Garcia et al., 2011; Garcia, Huffman, & Shimizu, 2010; Wallner, Aspernig, Millesi, & Machatschke, 2011; Wehrenberg & Dyrenfurth, 1983). Japanese macaques, for instance, face temperature as low as −20°C during the mating season (Hanya, Kiyono, & Hayaishi, 2007). These circumstances entail additional thermoregulatory costs along with mating activities, whereas food availability and daylight decrease (Garcia et al., 2011; Wehrenberg & Dyrenfurth, 1983).

In wild Japanese macaque groups, climate‐dependent food availability is closely linked to reproduction: Females come into oestrous more often after “high‐fruit” seasons (Takahashi, 2002) and produce more offspring after food resources during fall were abundant (Noma, Suzuki, & Izawa, 1998). In savanna baboons, females enter reproductive maturation earlier after rainfall periods related to increased resource availability (Bercovitch & Strum, 1993). In captive primate groups, or even in provisioned groups, the food supply can be kept quite stable throughout the year. These improved conditions can impact reproduction enormously: Birth rate increases (Takahata, Suzuki, Agetsuma, et al., 1998), infant mortality decreases, the onset of puberty is earlier and primiparous age is lower (Mori, Yamaguchi, Watanabe, & Shimizu, 1997; Watanabe, Mori, & Kawai, 1992). Still, reproduction in promiscuous primates has been shown to carry significant fitness costs even in an environment with abundant food and no predation (Hoffman et al., 2008). Furthermore, differences in AFR have been recognized in provisioned groups of macaques (Bercovitch, Lebron, Martinez, & Kessler, 1998; Silk, Clark‐Wheatley, Rodman, & Samuels, 1981), and females do not necessarily reproduce every year after entering sexual maturity (Koyama et al., 1992). In macaque groups where food availability is not decisive, factors such as social rank, habitat quality, and the socionomic sex ratio (i.e., the ratio of mature males to mature females within a group) have been used to explain differences in female reproductive performance (Fedigan, 1983; Harvey & Rhine, 1983; Koyama et al., 1992). Finally, ecological parameters such as photoperiod, rainfall patterns, and temperature have been considered (Cozzolino & Schino, 1998). No conclusive evidence, however, has been found to determine, which of the climatic variables plays a key role (Cozzolino & Schino, 1998; Cozzolino, Cordischi, Aureli, & Scucchi, 1992; Fedigan & Griffin, 1996; Gouzoules, Gouzoules, & Fedigan, 1981; Nozaki, Mori, & Oshima, 1990; Varley & Vessey, 1977).

While most of these studies focused on comparing different groups or groups that were translocated to different latitudes (see also review by Fooden & Aimi, 2005), less attention has been paid to the effect of meteorological circumstances within one group in which both provisioning and habitat remained stable (Cozzolino & Schino, 1998). Importantly, one key factor that could help females to improve fitness during the sexual maturation process has been largely neglected: The impact of sunshine. The potential of sunshine to produce essential vitamins is undisputed (Ashwell et al., 2010; Holick, 1995; Lambert, Reid, Kaye, Jennings, & Esler, 2002; Norman, 1998). Human and animal models underline the importance of vitamin D on female reproduction (for review see Janelle, Torrealday, Genevieve, & Lubna, 2012). Vitamin D deficiency delays puberty (Dicken et al., 2012) and reduces overall fertility (Halloran & Deluca, 1980). In endothermal animals, sun exposure is used to cover the appreciable energy costs to keep body temperature constant during winter months (Brown & Downs, 2007; Cui, Quan, & Xiao, 2006). In Japanese macaques, sunbathing is preferred over huddling and foraging during cold winter periods (Hanya et al., 2007; Kelley, Jablonski, Chaplin, Sussman, & Kamilar, 2016). On the basis of these behavioral preferences of thermoregulation, Hanya et al. (2007) proposed that the increment of skin temperature is higher after sunbathing than after huddling. Sunbathing is therefore considered to be an efficient strategy to improve energy balance and to cope with cold stress (Barrett, Gaynor, Rendall, Mitchell, & Henzi, 2004; Danzy et al., 2012). Both factors should promote successful maturation and rapid subsequent reproduction.

Taken together, in the northly distributed Japanese macaque, the energetic value of sunshine exposure, its stress‐reducing effect and its relation to essential vitamin procurement might help females to improve the physical condition required for sexual maturation—a period characterized by the costly body and hormonal changes essential for successful fertilization during the first gestation. We hypothesized that the amount of sunshine in the year females experienced the onset of puberty affected their primiparous age, with longer sunshine durations resulting in earlier AFR. The group under investigation was located in Austria, where the cold winter conditions are comparable to those in Japan. Because their translocation from Japan in 1996, births have occurred every year, and the amount of provisions has been incrementally adjusted yearly in accordance with the increase in group size. Census records have been kept continuously because the establishment of the park, documenting the number of births and deaths annually. This has yielded 20 years of data used in the present study to analyze female AFR in relation to the amount of sunshine in the year they should have entered puberty, taking other meteorological conditions (rainfall and temperature) and social parameters (group size and the socionomic sex ratio) into account (Cozzolino & Schino, 1998; Takahata, Suzuki, Okayasu et al., 1998).

## 2 METHODS

### 2.1 Ethical statement

This research adhered to the legal requirements of Austria and to the American Society of Primatologists Principles for the Ethical Treatment of nonhuman primates (see https://www.asp.org/society/resolutions/EthicalTreatmentOfNonHumanPrimates.cfm).

### 2.2 Study site and animals

The investigated Japanese macaques were kept under semi‐free conditions in the four‐hectare park “Affenberg Landskron” (Affenberg Zoobetriebsgesellschaft mbH, Carinthia, Austria; GPS: 46.6439014° 13.8994263°). The monkeys were brought to Austria in 1996 and derived from a wild group in Minoo, Osaka Prefecture, Japan (GPS: 34.6937° 135.50216°). The original group consisted of 23 females of different ages and 15 males no older than 4 years. In the following years (1997–September 1, 2016) the group size grew to 156 individuals (70 mature females≥3.5 years, 54 matured males≥4.5 years, 21 juveniles, and 11 infants born in 2016).

Every year from early April to late October, Affenberg Landskron opens its gates to visitors, who can walk through the enclosure within the scope of guided tours. Any interaction with the monkeys, such as feeding or touching, is strictly prohibited. Guided tours take visitors on a pathway, bordered on both sides with rope fences, through approximately one‐third of the park. The remaining two‐thirds of the enclosure provides a place of uninhibited retreat for the monkeys. The park is vegetated by a forest with deciduous and coniferous trees, natural undercover of bushes and grass as well as a small stream and a pond. This environment enables the monkeys to forage freely. To preserve the environment in the enclosure from over‐foraging, the monkeys are provisioned on a daily basis throughout the year. The daily provisions consist of mostly regional vegetables (mainly carrots and potatoes), fruits (mainly apples), and wheat. The food is spread over a wide area in the enclosure by the animal care staff twice a day (9 a.m. and 6 p.m.). Depending on the size of the group, the daily amount of food supplied is adjusted and estimated according to the average kcal required for each individual (Hanya, 2003; Hill, 1997). To control the group's population growth, tubal ligation was conducted on certain females. The first sterilization of females took place in 2000. Since then, a total of 67 females have been sterilized (2000: *N* = 2; 2001: *N* = 4; 2002: *N* = 13; 2003: *N* = 5; 2004: *N* = 0; 2005: *N* = 0; 2006: *N* = 2; 2007: *N* = 0; 2008: *N* = 0; 2009: *N* = 9; 2010: *N* = 5; 2011: *N* = 3; 2012: *N* = 4; 2013: *N* = 5; 2014: *N* = 0; 2015: *N* = 8; 2016: *N* = 7). Each of the sterilizations was performed before the onset of the mating season to avoid abortions. Tubal ligations were performed to avoid manipulating their mating behavior and physiology from the use of hormonal contraceptives. Particular attention was paid to ensure that existing matrilines remain intact. Females usually had raised two offspring before they were sterilized. Exceptions were made when the matriline was small. In those cases, females were not sterilized to prevent bloodline extinctions.

The animal care staff are able to identify all monkeys individually based on facial and other characteristics, from the beginning phase of the park's establishment. Since 1996, individual animal records have been taken by the Affenberg staff to document births, deaths, injuries, medical treatment (including sterilization records), and matrilineal family relationships of each individual. The Affenberg Landskron provided these records documenting a 20‐year period for the present study

### 2.3 Variables of interest

#### 2.3.1 Meteorological data

Villach (Carinthia, Austria), the study site's location, lies in the moderate temperature zone of central Europe. It has a continental climate with hot summers and cold winters. During winter the landscape is frequently covered with snow (https://en.climate‐data.org/europe/austria/carinthia/villach‐9338/). Comparable to the climate of Villach, the climate at the monkeys’ location of origin, Osaka Prefecture, is classified as warm and temperate with a significant rainfall. Lowest levels of precipitation occur during winter months (https://en.climate‐data.org/asia/japan/osaka‐prefecture/osaka‐1002/).

For the present study, meteorological data—sunshine duration (hr), temperature (°C), and rainfall (mm)—were obtained from the Central Institute for Meteorology and Geodynamics (ZAMG). The nearest ZAMG weather station (GPS: 46.6181° 13.8738°) is situated at 493 meters above sea level, which is about 140 m below Affenberg. Three individuals that entered our analyses were exposed to the Japanese climate during their year of puberty. In these cases, we used data from ZAMG for the period from August 1996 to December 2016 when the animals were already exposed to Carinthian climatic conditions. For the preceding period before their arrival from January 1995 to July 1996, we used data obtained from the Japan Meteorological Agency (JMA) weather station in Osaka (World Meteorological Organization [WMO] Station ID:47772, GPS: 34°40.9′N 135°31.1′E).

#### 2.3.2 Measure of meteorological data

The present study used data on sunshine duration (hr), temperature (°C), and rainfall (mm) provided online by the ZAMG and JMA weather stations. The following information on meteorological measurements was compiled online and via email correspondence with the ZAMG weather station: To measure sunshine duration (in hours) the weather stations used photocells to assess the differences between global radiation and diffuse sky radiation. If the differences (global radiation—sky radiation) exceeded a threshold of 120 W/m^2^, sunshine was detected. This adheres to WMO requirements. The sum of sunshine hr per month is provided online for public use.

To measure the daily air temperature (5 cm above ground) two observation times a day, that is 7 a.m. (T_07_) and 7 p.m. (T_19_) Central European Time were applied. This yielded a daily minimum (T_min_) and maximum (T_max_) air temperature. Subsequently, a monthly mean for each of the four quantities (₸_07_, ₸_19_, ₸_min_, ₸_max_) was used to calculate a monthly mean air temperature ${(〒}_{\text{monthly}}\;=\frac{{〒}_{07}+{〒}_{19}+{〒}_{\min}+{〒}_{\max}}{4}).$

The amount of rainfall was measured daily with a rain gauge (load cell) MPS TRWS 405/415. The monthly value equals the sum of rainfall (in mm) measured for each day of that month.

For more details on meteorological data see:

ZAMG: https://www.zamg.ac.at/cms/en/climate/climate‐overview and JMA weather station in Osaka (WMO Station ID:47772): https://www.data.jma.go.jp/obd/stats/etrn/view/monthly_s3_en.php?block_no=47772&view=1.

#### 2.3.3 Year of puberty onset and sexual maturation

To investigate the influence of annual sunshine duration on female sexual maturation and subsequent AFR, the onset of puberty had to be estimated. Due to the lack of long‐term‐hormone data, we used an age‐based approach rather than direct measurement of the exact onset of puberty of each individual. We estimated the onset of puberty and sexual maturation based on published data as well as on reproductive data of the study group: Female Japanese macaques typically engage in sexual activities at an age of 3.5 years. This age corresponds to a sustained increase in luteal progesterone followed by estrous behavior and first (although not necessarily successful) ovulation (Fooden & Aimi, 2005; Fujita, Sugiura, Mitsunaga, & Shimizu, 2004; Gunst, Leca, & Vasey, 2015; Leca, Gunst, & Vasey, 2014; Nakamichi & Yamada, 2010; Wolfe, 1978). More in‐depth studies on underlying neuroendocrine changes during puberty are available for rhesus macaques, a closely related species with a life history comparable to that of Japanese macaques (see Shimizu, 2008 for comparable hormone pattern). A review about environmental and social influences on neuroendocrine puberty in rhesus macaques refers to the onset of puberty as “the increased activation of the hypothalamic–pituitary–gonadal axis as a result of increased gonadotropin‐releasing hormone (GnRH) release” (Stephens & Wallen, 2013). This initial activation of female sex hormones occurs as early as 24 months of age but does not yet lead to successful ovulation. In the present study, we refer to the year of puberty onset as the year (January–December) in which females are approx. between 18 and 30 months old. Within this preovulatory period, we expected environmental conditions to affect female subsequent maturation and, thus, their AFR (see below).

In the investigated group, 36% of the females gave birth at 4 years of age: Those females clearly exhibited successful ovulation during the mating period when they reached the age of 3.5 years. We, therefore, refer to the earliest year of sexual maturation as the year (January–December) in which females reached the age of 3.5.

#### 2.3.4 Age at first reproduction

AFR equates to a female's age on the date she was observed with an offspring for the first time, irrespective of whether the offspring was alive, dead, or died shortly after birth. The semi‐free ranging, vegetated conditions of the enclosure offered the opportunity for animals to withdraw from human encounters. No births have been observed to date. Stillbirths, abortions, or miscarriages could therefore not be considered in the present study.

### 2.4 Data processing and control variables

The present study investigated the influence of meteorological parameters of the year in which females theoretically experienced the onset of puberty (year of puberty onset; see Section 2.3) on their AFR. Therefore, monthly meteorological data obtained from the weather station (see Section 2.3) was used to calculate yearly means (January–December) of sunshine duration (hr), temperature (°C), and rainfall (mm).

#### 2.4.1 Meteorological parameters during the earliest year of sexual maturation

To control for any additional influence of environmental circumstances during the earliest year of sexual maturation (see Section 2.3), meteorological conditions during the year females reached the aged of 3.5 entered our analyses as control variables (Fooden & Aimi, 2005; Fujita et al., 2004; Leca et al., 2014; Wolfe, 1978). Again, yearly means (January–December) of sunshine duration (hr), temperature (°C), and rainfall (mm) were used.

#### 2.4.2 Social parameters during the earliest year of sexual maturation

Due to their possible influence on reproduction as described in previous studies on nonhuman primates, we controlled for social circumstances (group size and socionomic sex ratio; Cozzolino & Schino, 1998; Takahata, Suzuki, Okayasu, et al., 1998; van Noordwijk & van Schaik, 1999). Group size of each year refers to the number of individuals reported as being alive at the onset of each mating season irrespective of their sex and age. The socionomic sex ratio (Cozzolino & Schino, 1998) of each year was calculated as the ratio of mature males to mature females $(\frac{\text{No}\;.of\,\text{mature males}}{\text{No}\;.of\,\text{mature females}}\times 100).$ Mature animals were defined as those individuals who already reached sexual maturity according to their age, that is, ≥4.5 years for males and ≥3.5 years for females (Takahata, 1980; Wolfe, 1978). Both parameters (group size and socionomic sex ratio) were measured at the onset of the mating period (1 September) in which females turned 3.5 years and had their first opportunity to conceive an offspring (earliest year of sexual maturation; see Section 2.3).

### 2.5 Statistics

Partial least square (PLS) regression was applied to investigate the relative importance of meteorological and social parameters during the year of puberty onset (see Section 2.3) and the earliest year females could have reached sexual maturation (see Section 2.3.2) on females’ AFR. PLS regression is especially suited for datasets with small sample size and high collinearity (Carrascal, Galván, & Gordo, 2009). We included only females who reached sexual maturity at the Affenberg Landskron. Females who were already sexually matured when the group arrived on site were excluded. The selection of the latent variables in the fitting of a PLS regression model is critical, and the predictive power of the model was evaluated using cross‐validation (Wold, Ruhe, Wold, & Dunn, 1984). The prediction accuracy of the models was tested by using the *R* ^2^ of the calibration and validation set and the root mean square error (RMSE) of calibration and validation. The initial model contained eight predictors (RMSEP= 0.80 and *R* ^2^= 0.15), whereas the final model contained only four predictors (RMSEP= 0.79 and *R* ^2^= 0.19). An accurate and stable model is indicated by a lower RMSE and a higher *R* ^2^ (Vibhute, Kale, Mehrotra, Dhumal, & Nagne, 2018). In PLS regression plots, variables located on the perimeter of the circle are strongly correlated. Variables close to each other are positively correlated and variables that are far apart are negatively correlated (Kabir et al., 2017). The beta/standardized coefficient test was performed to evaluate the effect of each predictor on the dependent variable (AFR). Variables of significance (*p* < .05) show a coefficient different from 0.

All descriptive statistical analyses were carried out using R version 3.2.2 (The R Foundation for Statistical Computing, Vienna, Austria, http://www.r‐project.org). The partial least squares regression was performed using Unscrambler 10.4.1 software (CAMO AS, Oslo, Norway).

## 3 RESULTS

### 3.1 Descriptives

In 20 birth seasons (1997–2017), a total of 206 infants were born: 102 males, 101 females (Figure S1) and three of unknown sex. Of these, 20 infants (10 males and seven females and three of unknown sex) were reported to have died during their first half year of life. All births occurred between February and August and mainly in late April and throughout May. Only five births took place in February, July, and August (Figure S2). In each birth season, between one and 15 infants were born (Figure S1). Given a 172‐day gestation period (Fooden & Aimi, 2005), we can assume that most infants were conceived in late October and throughout November (Figure S3). In total, 74 females gave birth for the first time at Affenberg Landskron. The youngest female gave birth to her first offspring at the age of 3.03 years, the oldest at 7.86 years. The sample included 13 mothers with late reproduction (defined as mean AFR plus one standard deviation [*SD*]) out of which five were born in Japan and relocated 1996 to Affenberg Landskron. The other eight were born at the Affenberg Landskron.

The meteorological parameters cover the years 1995–2016. The yearly mean sunshine duration ranged from 143.89 to 202.67 hr (mean = 178.66 hr; *SD* = ±14.03 hr), the yearly mean temperature ranged from 8.70 to 16.60°C (mean = 9.95°C; *SD* = ±1.67°C), and the yearly mean rainfall ranged from 67.58 to 132.67 mm (mean = 98.12 mm; *SD* = ±16.85 mm). For further descriptive statistics see Table 1 (also see Figures S4–S6).

**Table 1:** Descriptive statistics of years under investigation (1995–2016): Females’ first parity, sunshine duration in the year of puberty and controlling variables

Variable	N	Min.	Q1	Median	Q3	Max.
Age (in years) at first reproduction	74	3.03	4.10	4.96	5.08	7.86
Sunshine duration (hr) year of puberty onset	74	143.90	170.01	185.70	189.70	202.70
Temperature (°C) year of puberty onset	74	8.70	9.43	9.78	9.92	16.60
Rainfall (mm) year of puberty onset	74	67.58	85.58	102.08	108.25	132.67
Sunshine duration (hr) year of sexual maturation	74	143.90	170.10	178.00	185.90	202.70
Temperature (°C) year of sexual maturation	74	8.70	9.01	9.64	9.82	12.18
Rainfall (mm) year of sexual maturation	74	67.58	86.83	101.04	110.38	132.67
Group size^a^	74	36.00	83.00	109.00	141.00	158.00
Sex ratio^a^	74	12.50	58.62	70.59	75.86	85.37

*Note*: Given are measures of central tendency and variance of the distribution. Year of puberty onset: The year females reached the age of 2.5 and theoretically entered puberty. Year of sexual maturation: The earliest year of sexual maturation, i.e., when females reached the age of 3.5. Given are the minimum (Min.) and maximum (Max.) value, the first (Q1) and third (Q3) quantile, and the median. Meteorological data from 1995 to 2016 were provided by the JMA weather station in Osaka and the ZAMG. Birth records from 1997 to 2017 were provided by the Affenberg Zoobetriebsgesellschaft mbH.

^a^ Measured at the onset of the mating period (1 September) in which females turned 3.5 years.

**Figure 1:**
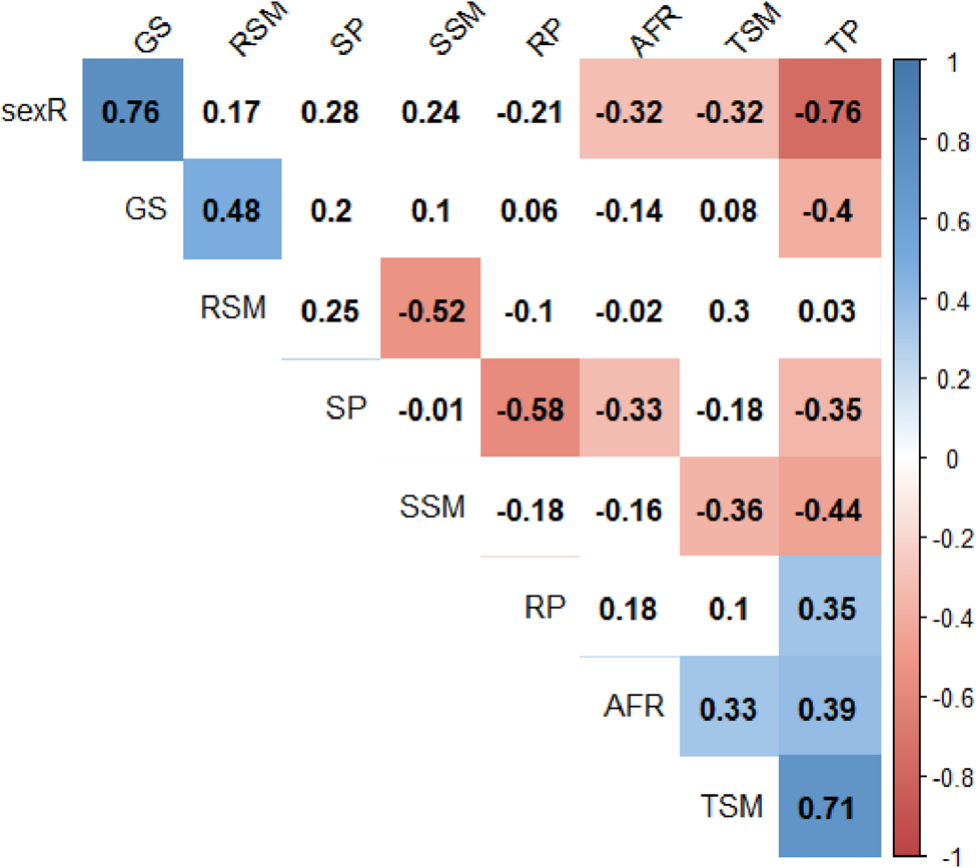
Pairwise correlation between initial variables of interest. Descriptive statistics (correlation matrix; *N* = 74) of variables included in the initial model: GS=group size, sexR=socionomic sex ratio, RSM=mean rainfall (mm) in the earliest year of sexual maturation, SP=mean sunshine duration (hr) in the year of puberty onset, SSM=mean sunshine duration (hr) in the earliest year of sexual maturation, RP=mean rainfall (mm) in the year of puberty onset, AFR=age at first reproduction, TSM=mean temperature (°C) in the earliest year of sexual maturation, TP=mean temperature (°C) in the year of puberty onset. All predictors of the final model (sexR; SP; TSM; TP) were significantly (*p* < .001) correlated with females’ age at first reproduction. Positive correlations are highlighted in blue, negative correlations in red

Figure 1 shows the pairwise correlation between the initial variables of interest. All the predictors of the final PLS regression model were significantly (*p* < .001) correlated with AFR. Among them, mean sunshine duration in the year of puberty onset had the highest negative correlation with AFR (*r *= −0.33, *p* < .001). The mean temperature during the year of puberty onset had the highest positive correlation with AFR (*r* = 0.39, *p* < .001).

### 3.2 Effects of metrological and social factors on AFR

To detect outliers and influencers in our dataset, we carried out a Hotelling T^2^ statistic, a multivariate generalization of the student *t* test (Hotelling, 1933). All samples were kept to best define the model space.

**Figure 2:**
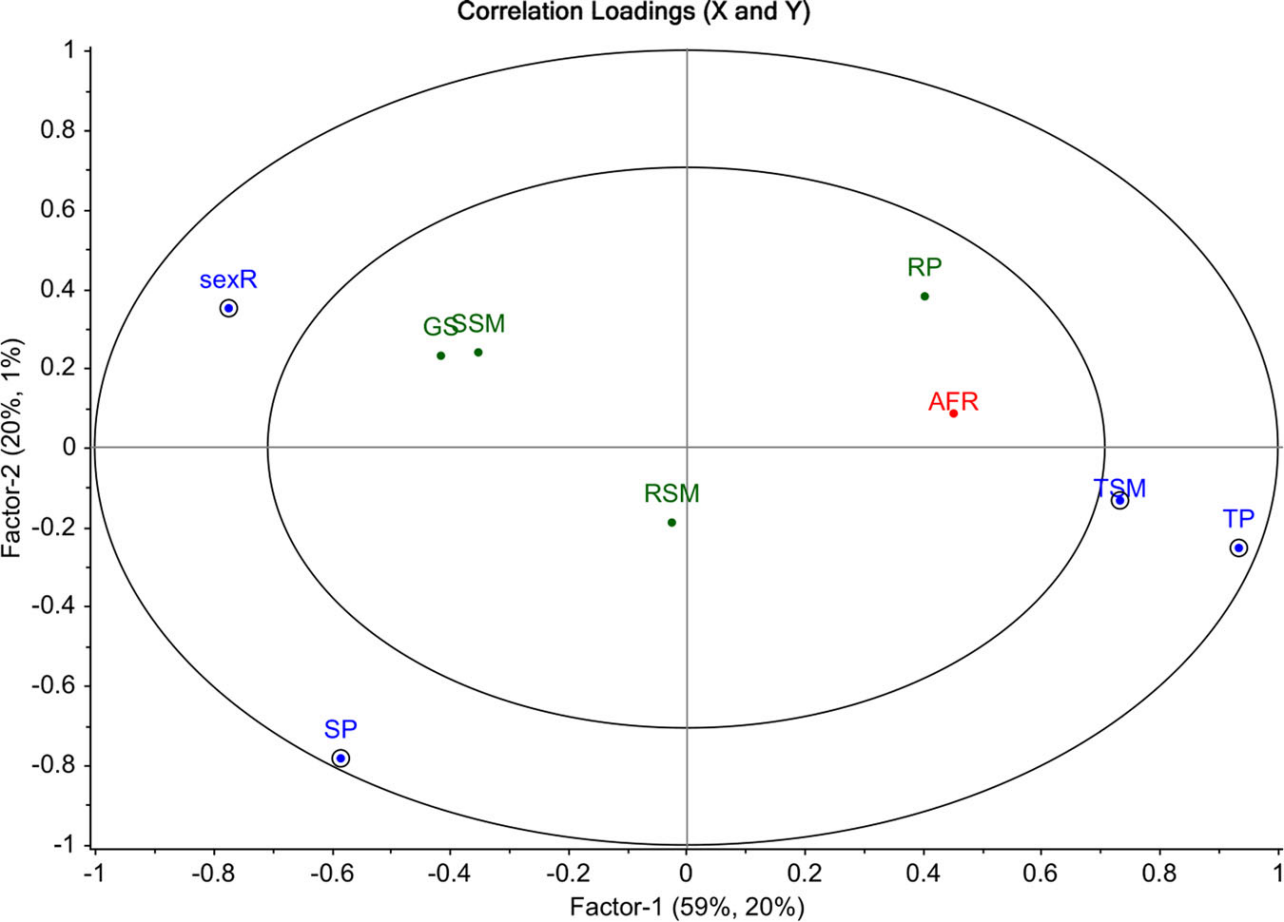
Correlation circle of partial least squares (PLS) regression showing the correlations of the variables with the first two components. Predictor variables that contributed to the final PLS regression model are illustrated as blue dots in the outer circle of the correlation circle: SP=mean sunshine duration (hr) in the year of puberty onset, sexR=socionomic sex ratio, TSM=mean temperature (°C) in the earliest year of sexual maturation, TP=mean temperature (°C) in the year of puberty onset. The dependent variable is illustrated as a red dot: AFR=age at first reproduction. For other abbreviations see Figure1

**Figure 3:**
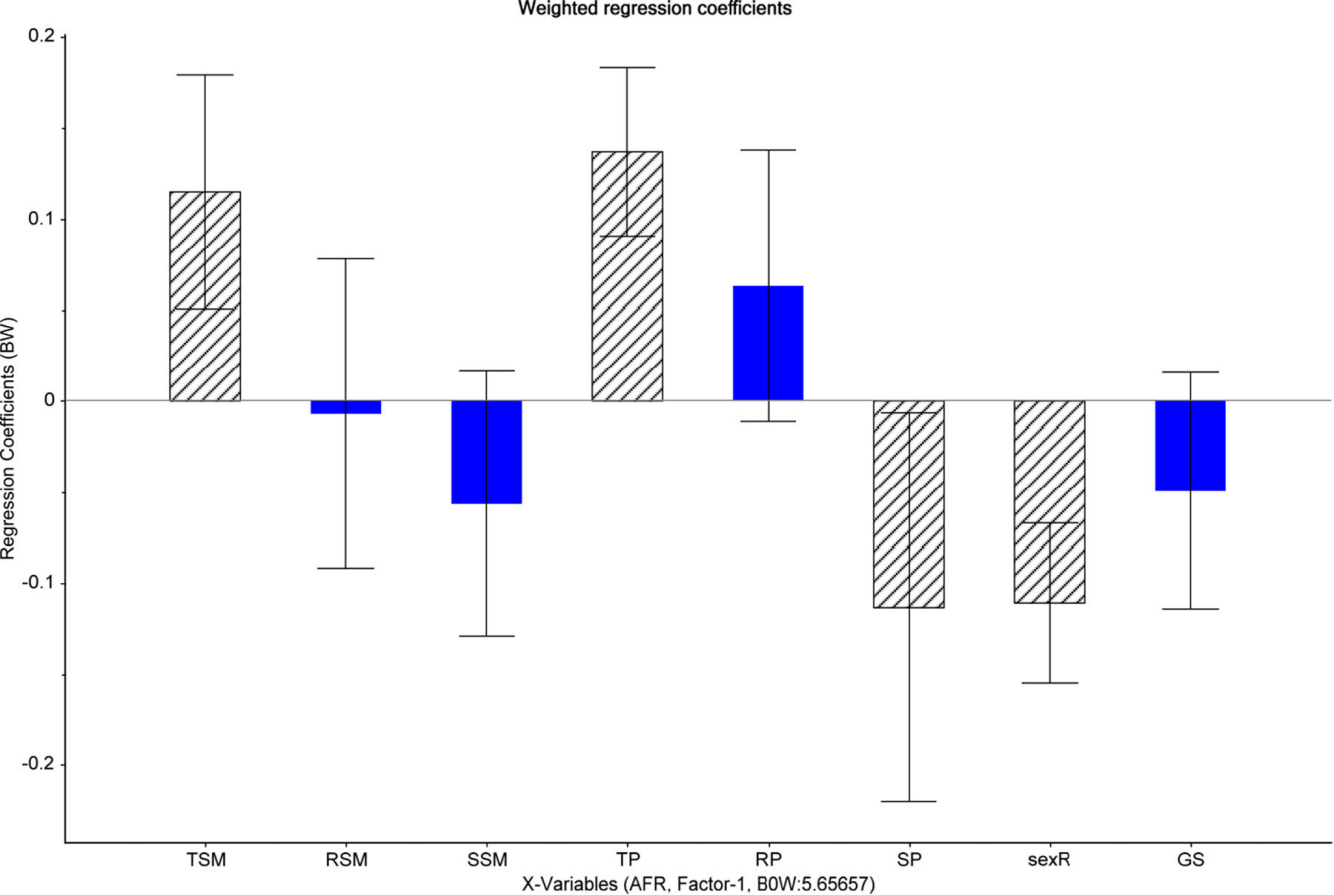
Plot of the regression coefficient test showing the effect of each predictor on the dependent variable age at first reproduction (AFR). Predictor variables of the final partial least squares (PLS) regression model are represented as dashed bars with a coefficient different from 0: SP=mean sunshine duration (hr) in the year of puberty onset, sexR=socionomic sex ratio, TSM=mean temperature (°C) in the earliest year of sexual maturation, TP=mean temperature (°C) in the year of puberty onset. SP and sexR were negatively, TSM and TP positively correlated with the dependent variable AFR. For other abbreviations see Figure1

In the PLS regression model, AFR was mainly explained by Component 1 (20%) and to a lesser extent by Component 2 (1%). The correlation plot (Figure 2) revealed a significant influence of sunshine duration on female AFR: If the mean duration increased in the year of puberty onset, AFR decreased. In other words, females who had their (theoretically expected) onset of puberty in years with more sunshine, reproduced for the first time at significantly younger ages than females who experienced less sunshine at that stage of development. The following variables additionally entered the final PLS regression model as important control variables: Mean temperature in the year of puberty onset, mean temperature in the year of sexual maturation and socionomic sex ratio (all variables important for the final model are represented as blue dots in the outer circle of Figure 2). The mean temperature in both years showed a positive correlation with females’ AFR, whereas socionomic sex ratio was negatively correlated with females’ AFR. Those variables are represented in Figure 3 as dashed bars with a coefficient different from 0. The remaining variables (sunshine duration in the year of sexual maturation, rainfall in the year of puberty onset, rainfall in the year of sexual maturation and group size) appeared to be not decisive for our final model (represented as green dots in the inner circle of Figure 2; blue bars in Figure 3 with coefficients not different from 0).

## 4 DISCUSSION

The present study investigated the influence of meteorological conditions on female AFR in a semi‐free‐ranging group of Japanese macaques. Whereas previous studies focused on the influence of temperature, rainfall, and photoperiod on reproductive patterns in macaques (e.g., Cozzolino et al., 1992; Fedigan & Griffin, 1996; Varley & Vessey, 1977), we hypothesized that increased sunshine duration in the year females enter puberty would be of benefit for early reproduction. In line with our predictions, our results revealed that a longer sunshine duration in the year of puberty onset lowered female AFR significantly.

Sexual maturation (measured by a sustained increase in luteal progesterone and first successful ovulation) is estimated to occur at the earliest at the age of 3.5 when females begin to experience estrus and start to engage in sexual activities (Leca et al., 2014; Stephens & Wallen, 2013; Wolfe, 1978). This is in line with our data showing that most females conceived their first offspring in the year or after the year they turned 3.5 years old. Sexual maturation is preceded by a crucial shift in hormonal and bodily functions to initiate the transition from juvenile to adult life stage. This developmental period is specifically prone to environmental factors that influence the timing of sexual maturation (Leca et al., 2014; Stephens & Wallen, 2013). The present study reports that sunshine duration during the period of about 18–30 months of age is relevant in terms of early reproduction in female Japanese macaques.

But how could sunshine influence sexual maturation and subsequent reproduction? Here we discuss three effects of sunshine that might play a role for sexual maturation in the northerly distributed Japanese macaques: (a) Its contribution to energy status (b) reduction of cold stress, and (c) synthesis and maintenance of vitamins sensitive to ultraviolet (UV) radiation.

First, during the age in which primates experience the onset of puberty, energy resources are still required for growth and maintenance. This makes sexual maturation an additional burden (Stephens & Wallen, 2013). Although the direct use of sunshine has not been considered in previous studies dealing with reproductive performance in macaques, the role of females’ physiological condition has often been examined. Garcia et al. (2011), for instance, state that “physiological modulation of reproductive outcome in females starts with mechanisms that adjust the probability of conception in response to maternal energy availability.” Their findings suggest that females with a higher energy status are more likely to afford early and rapid reproduction. How beneficial climatic conditions in terms of resource availability and related energy resources positively affect reproductive maturation has also been shown in baboons (Bercovitch & Strum, 1993). Besides huddling and forming clusters (Eppley, Watzek, Hall, & Donati, 2017; Hori, Nakayama, Tokura, Hara, & Suzuki, 1977; Zhang & Watanabe, 2007), sunbathing has been generally discussed as a thermoregulatory strategy in primates to improve energy status (Barrett et al., 2004; Danzy et al., 2012).

Second, the literature on human and nonhuman primates ascribe the timing of puberty to be particularly susceptible to environmental circumstances such as psychosocial stress (Blomquist, 2009a). Due to their despotic social system and their northern distribution, Japanese macaques have to cope with both cold and social stress particularly during the mating season, which occurs in fall and winter. A recent study on female Japanese macaques in the Jigokudani Monkey Park reported that bathing in thermal hot springs is an alternative thermoregulatory strategy in this particular group (Takeshita, Bercovitch, Kinoshita, & Huffman, 2018): The direct heat of the hot water is a physiological benefit because it significantly reduces the glucocorticoid levels of female monkeys. A stress‐induced increase in glucocorticoid levels in different animal species including macaques impairs the secretion of sexual hormones (e.g., GnRH, luteinizing hormone [LH], estrogen) important for sexual maturation and cycling (Hayashi & Moberg, 1989; Oakley et al., 2008, Rivier & Rivest, 1991). In our study, greater access to sunbathing opportunities during the year of puberty might have reduced cold and social stress more efficiently, yielding a physiological benefit in terms of female sexual maturation. The importance of sunlight is supported by distinctive behavioral and morphological features ascribed to this species in relation to the sun: The body posture of Japanese macaques tends to be more curled when the temperature is lower (Hanya et al., 2007). When the weather is clear, however, Japanese macaques straighten their posture to expose more body surface to the sun; this is preferred over huddling (Hanya et al., 2007; see Kelley et al., 2016 for similar findings in *Lemur catta*) and feeding (personal observations). Japanese macaques of the Arashyama B group (Kyoto, Japan) were observed to choose the eastern‐most facing slope next to the provisioning grounds as their sleeping site during winter. At first light in the morning, they appeared on the exterior branches sitting up straight, exposing the front of their bodies to the early warm sunshine, before coming down to the snow‐covered ground. In other times of the year they choose alternative parts of their home range, for example, in summer cooler and less mosquito‐affected locations (Huffman, personal observations, 1979–1980; see Cui et al., 2006 for similar observations on black and white snub‐nosed monkeys). During the daytime, we also repeatedly saw sunbathing in this particular group. Sunbathing is generally performed by lying in the sun or sitting upright with the belly and chest exposed to the sun in wind‐protected areas. Hanya et al. (2007) proposed that the increment of skin temperature is higher after sunbathing than after huddling, which underlines the effectiveness of sunbathing as a thermoregulatory strategy.

Third, the exposure to direct sunlight via sunbathing activities might also help to extract the UV components of the sun. The sun is the major source of vitamin D, an essential vitamin whose synthesis starts in the skin when exposed to UV radiation (Engelsen, 2010). In Japanese macaques, the body parts exposed to the sun via sunbathing show less hair growth than other body parts, and the visible skin underneath has a partly blue color. So far, the literature has not forwarded any explanation as to why Japanese macaques have reduced fur density and darkened the skin in this area (see Erickson & Montagna, 1975 for a detailed review on the description of pigmentation in rhesus macaques). Perhaps these anatomic features help to adapt physiologically and behaviorally (change of body position) to variable climate conditions. This assumption has been previously made for vervet monkeys (Danzy et al., 2012) and ring‐tailed lemurs (Chaplin et al., 2014). In the latter, the pigmentation of the ventral body part, directed towards the sun during sunbathing, is expected to increase the benefit of sunbathing because it maximizes solar exposure and passive warming of the body (Chaplin et al., 2014, also see Terrien, Perret, & Aujard, 2011). In humans, solar exposure of darkly pigmented skin is expected to improve energy absorption and to help balance the maintenance of vitamin D and the B vitamin folate (Jones, Lucock, Veysey & Beckett, 2018). Both UV‐sensitive vitamins are important for sexual maturation and fetal development in human and animal models (Jablonski & Chaplin, 2000; Janelle et al., 2012; Jones, Lucock, Veysey, & Beckett, 2018). Although our study does not provide sufficient data to support any relation between skin pigmentation and sunbathing in Japanese macaques, we strongly recommend future research to investigate sunbathing activities along with anatomic features and the synthesis of UV‐related vitamins important for sexual maturation and subsequent successful reproduction.

Despite the annual differences in sunshine duration measured in the present study, the investigated group experienced regular seasonal changes in temperature and photoperiod length comparable to their original homeland in Japan. On the basis of the 20‐year dataset, the onset of mating always took place in September, which is in line with data on free‐ranging groups (Fooden & Aimi, 2005; Fujita et al., 2004). Comparable to Japan, except for summer, the direct use of sunshine is rather rare due to a high frequency of cloudy, rainy, or snowy days. Particularly during cold winter days, lengthier sunshine durations might be of physiological benefit. We are aware that our study did not measure individual differences in body condition, energy balance, quality of ovarian cycling, social factors such as social rank or genetic predisposition (Blomquist, 2009a, 2009b; Blomquist, 2012; Garcia et al., 2010). Rank‐related food availability was not an issue here because the group was provisioned evenly around the enclosure and the amount of provisioned food was adjusted to increasing group size every year. Furthermore, individuals were not confronted with particularly stressful or life‐threatening events such as intergroup encounters or predation. Hence, besides climatic differences, the only variability between years was growing group size and the socionomic sex ratio, both of which we controlled for in our models. The former had no effect on female AFR: The values did not increase or decrease over time. Nonetheless, in those mating seasons when the number of matured males converged with the number of matured females, the AFR appeared to be lower than in mating seasons when matured males were scarce.

In conclusion, our study provides the first insight that natural sunshine duration influences AFR of female nonhuman primates. Whereas our study provides plausible explanations for how sunshine duration could interact with a female's reproductive development, the underlying physiological processes were not measured. This calls for future studies to reveal how sunshine helps to improve body functions needed for an early onset of reproduction. Because the individual point of maturation is difficult to determine in (semi‐) free‐ranging groups, the present study refrained from dividing the years under investigation into further intervals. Continuous measurements of a female's physiological state (e.g., GnRH, LH, FSH, estrogen, and cortisol levels) would yield more detailed information on individual differences in the timing of sexual maturation in relation to sunbathing behaviors. These measurements would furthermore provide the opportunity to examine age at first conception and to determine whether it differs from AFR.

## CONFLICT OF INTERESTS

The authors declare that there are no conflict of interests.

## Supporting information

Supporting information

Supporting information

Supporting information

Supporting information

Supporting information

Supporting information
