# Supplementary material for: On the sunny side of (new) life: Effect of sunshine duration on age at first reproduction in Japanese macaques (Macaca fuscata)
Source: Am J Primatol. 2019 Jun 27;81(7):e23019. doi: 10.1002/ajp.23019 (PMC6773204; doi:10.1002/ajp.23019)
Supplement: Supplementary file 1 — Supporting information [file AJP-81-na-s001.pdf]

Figure S1 Total number of male and female infants per birth season

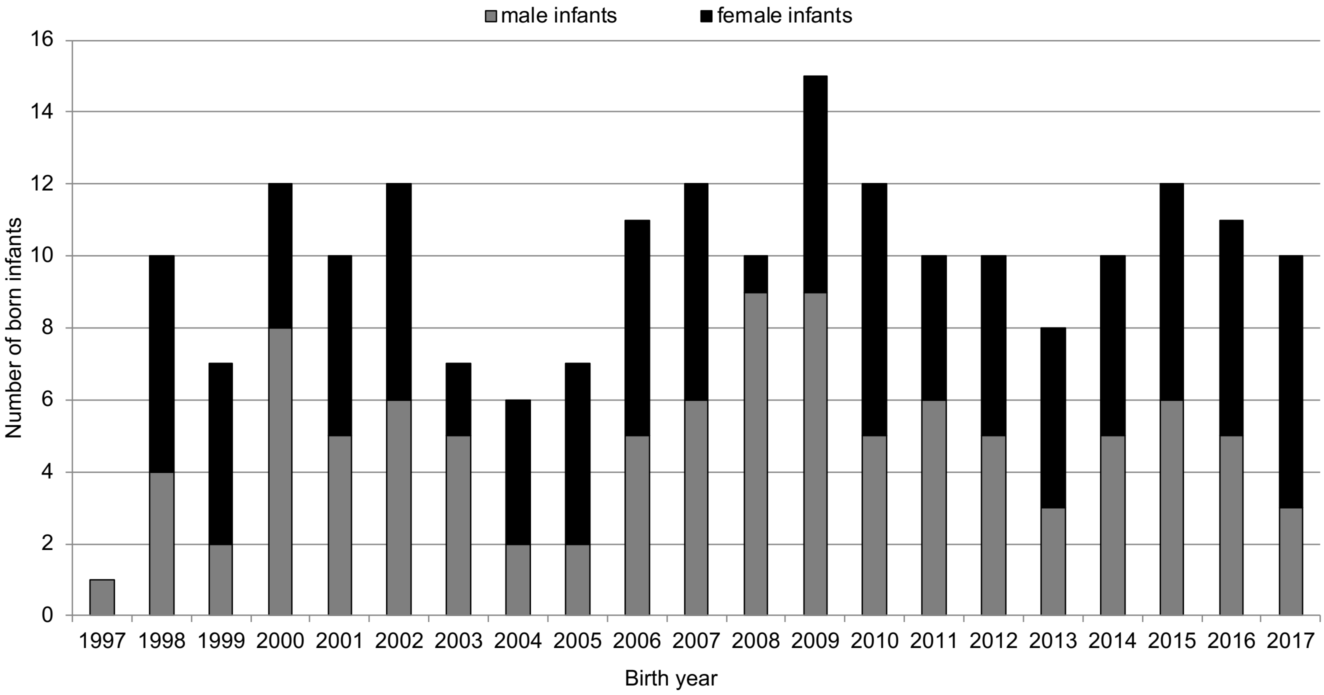

Male and female infants born per year within the 20 years observation period (in total N= 203, three infants were excluded due to unknown sex).
