# Supplementary material for: On the sunny side of (new) life: Effect of sunshine duration on age at first reproduction in Japanese macaques (Macaca fuscata)
Source: Am J Primatol. 2019 Jun 27;81(7):e23019. doi: 10.1002/ajp.23019 (PMC6773204; doi:10.1002/ajp.23019)
Supplement: Supplementary file 2 — Supporting information [file AJP-81-na-s002.pdf]

Figure S2 Birth timing of infants born during the study period

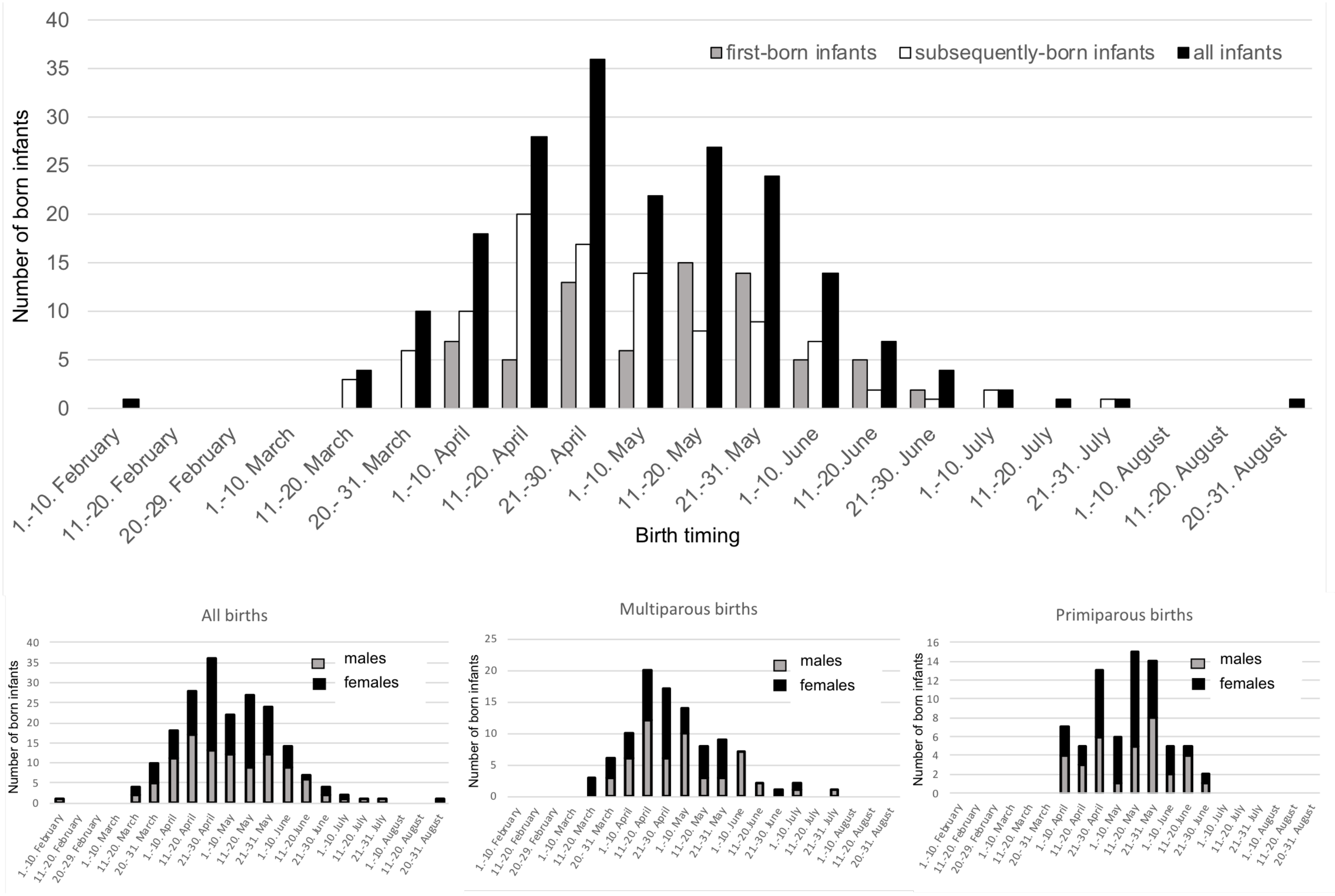

Upper panel: birth timing of infants born within the 20 years observation period. Infants are categorized as first- and subsequently-born infants (in total N=204, two infants were excluded due to unknow day of birth). Lower panel: Birth timing of male and female infants within the 20 years observation period (left), additionally categorized into first- (right) and subsequently-born (middle) infants (in total N= 201, two infants were excluded due to unknown day of birth, three infants were excluded due to unknown sex).
