# Supplementary material for: On the sunny side of (new) life: Effect of sunshine duration on age at first reproduction in Japanese macaques (Macaca fuscata)
Source: Am J Primatol. 2019 Jun 27;81(7):e23019. doi: 10.1002/ajp.23019 (PMC6773204; doi:10.1002/ajp.23019)
Supplement: Supplementary file 3 — Supporting information [file AJP-81-na-s003.pdf]

Figure S3 Estimated conception date of infants born in the study period

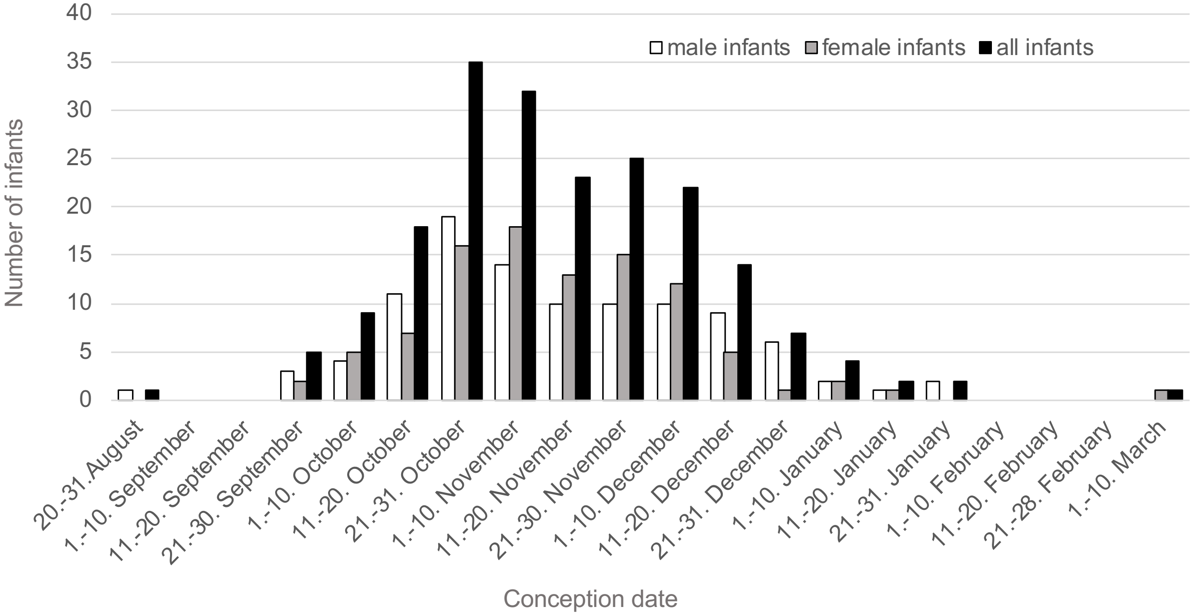

Estimated conception date of infants born within the 20-years observation period. Conception date was calculated according to a 172-day gestation period (Fooden & Aimi, 2005). In total N=204 (two infants were excluded due to unknow day of birth).
