# Supplementary material for: On the sunny side of (new) life: Effect of sunshine duration on age at first reproduction in Japanese macaques (Macaca fuscata)
Source: Am J Primatol. 2019 Jun 27;81(7):e23019. doi: 10.1002/ajp.23019 (PMC6773204; doi:10.1002/ajp.23019)
Supplement: Supplementary file 5 — Supporting information [file AJP-81-na-s005.pdf]

Figure S5 Annual differences in mean temperature (°C)

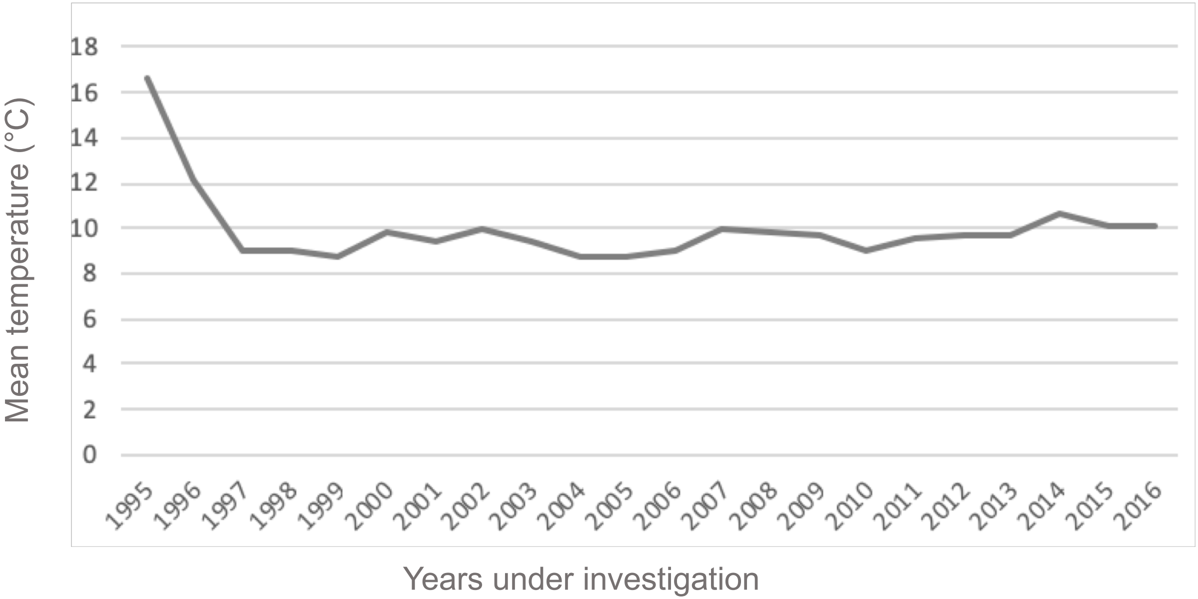

Differences in yearly mean temperature (°C) investigated prior to females' first reproduction from 1995 to 2016. Three females under investigation were exposed to the Japanese climate during their year of puberty. For the preceding period prior to their arrival from January 1995 to July 1996 we used data obtained from the Japan Meteorological Agency (JMA) weather station in Osaka (WMO Station ID:47772, GPS: 34°40.9'N 135°31.1'E). Data from Central Institute for Meteorology and Geodynamics (ZAMG) was used for the period from August 1996 to December 2016 when the animals were already exposed to Carinthian climatic conditions.
